# Effects of microRNA-126 on cell proliferation, apoptosis and tumor angiogenesis via the down-regulating ERK signaling pathway by targeting EGFL7 in hepatocellular carcinoma

**DOI:** 10.18632/oncotarget.17283

**Published:** 2017-04-20

**Authors:** Cheng Gong, Jing Fang, Guang Li, Han-Han Liu, Zhi-Su Liu

**Affiliations:** ^1^ Department of General Surgery, Research Center of Digestive Diseases, Zhongnan Hospital of Wuhan University, Wuhan 430071, P.R. China; ^2^ Department of Oncology, Wuhan Pu-Ai Hospital, Tongji Medical College, Huazhong University of Science and Technology, Wuhan 430034, P.R. China; ^3^ Department of Pathology, Maternal and Child Health Hospital of Hubei Province, Wuhan 430070, P.R. China

**Keywords:** microRNA-126, epidermal growth factor-like domain 7 (EGFL7), hepatocellular carcinoma, ERK signaling pathway, proliferation

## Abstract

This study intends to explore the effects of microRNA-126 (miR-126) on cell proliferation, apoptosis, and tumor angiogenesis in hepatocellular carcinoma (HCC) by regulating epidermal growth factor-like domain 7 (EGFL7) through extracellular signal-regulated kinase (ERK) signaling. HCC tissues and adjacent normal tissues were obtained from 184 HCC patients. HCC cells were separately transfected with recombinant plasmids. Western blotting and qRT-PCR were applied to detect miR-126 and EGFL7, ERK, Fas/FasL, Bcl-2, Caspase mRNA and protein levels. CCK8 and TUNEL were performed to determinate cell proliferation and apoptosis. Flow cytometry was used to analyze cell cycle distribution. Rats model of HCC was constructed, and tumor weight and the number of new blood vessels were recorded after 3 weeks of tumor transplantation. Compared with the adjacent normal tissues, HCC tissues exhibited lower miR-126 expression, and higher EGFL7, and ERK mRNA and protein levels. Overexpression of miR-126 in HCC cell lines suppressed EGFL7, ERK, Bcl-2, and P-ERK, and increased apoptotic-associated proteins Fas/FasL and Caspase-3, and it inhibited cell proliferation and induced cell apoptosis. Overexpression of miR-126 in nude mice resulted in reduced tumor weight and less new blood vessels in tumors. The inhibition of miR-126 decreased cell apoptosis, and enhanced cell proliferation and tumor angiogenesis. This study demonstrates that miR-126 might decrease cell proliferation, induce apoptosis, and inhibit tumor angiogenesis in HCC by inhibiting EGFL7 via down-regulating the ERK signaling pathway.

## INTRODUCTION

Hepatocellular carcinoma (HCC) is the sixth most common cancer and the third leading cause of cancer mortality worldwide [1]. Each year, over 110, 000 deaths are estimated among patients suffering from HCC in China [2]. Although modern therapeutic strategies are relatively effective, HCC patients have an unsatisfactory prognosis owing to tumor metastasis and intrahepatic recurrence [3]. The morbidity and mortality of HCC are still increasing [4]. In spite of more comprehensive understanding of HCC development and progression, it remains important to determine the molecular mechanisms in HCC in order to provide more effective targeted therapies. MicroRNAs (miRNAs) are a family of endogenously expressed, small non-coding RNAs which modulate gene expression by binding to the 3′ untranslated region (3′-UTR) of their target mRNAs, leading to repression or degradation of protein translation [5]. miRNAs functions in various physiological processes, including cell proliferation, differentiation, apoptosis, invasion, metastasis metabolism, and maturation. Their aberrant expression may modulate these pathological processes in human cancers through altered regulation of critical oncogenes or tumor suppressors [6, 7]. The abnormal expressions of miRNAs have been presented in HCC to affect the development and progression [8–10].

miR-126 is dysregulated in many human cancers, modulating the proliferation, migration, and invasion of cancer cells in conjunction with various target genes [11–13]. miR-126 serves as a tumor suppressor, inhibiting cell proliferation and inducing cell apoptosis in HCC [14]. Saito *et al*. reported that miR-126 is situated within the gene for epidermal growth factor-like domain 7 (EGFL7), which is highly expressed in vascularized tissues and endothelial cells and acts as an important gene in angiogenesis [15]. Angiogenesis is dysregulated in chronic liver disease and plays a crucial role in the early stages of tumor development [16–18]. In addition, extracellular signal-regulated kinase (ERK) is reported to contribute to cell proliferation in HCC [4]. Our study aims to explore the potential mechanism of miR-126 affecting cell proliferation, apoptosis, and angiogenesis in HCC via mediating EGFL7 and the ERK signaling pathway.

## RESULTS

### Correlation between clinicopathological characteristics and the prognosis of HCC patients

One hundred eighty-four HCC patients including 156 males and 28 females, with a mean age of 48.97 ± 9.33 years (aged from 23–72 years) were enrolled in our study. They had not received any treatment against primary tumors before surgery. They had no other complications or family disease history, and they received adjuvant three-dimensional conformal radiation therapy (3DCRT) after surgery. According to tumor-node-metastasis (TNM) staging [19], there were 63 cases with stage I HCC, 12 cases with stage II HCC, 61 cases with stage III HCC, and 48 cases with stage IV HCC. There were 31 cases without liver cirrhosis, 46 cases with mild liver cirrhosis, 72 cases with moderate liver cirrhosis, and 35 cases with severe liver cirrhosis. Regarding the differentiation degree, there were 38 cases with grade I; 66 cases with grade II; 33 cases with grade III; and 47 cases with grade IV. During follow up, 68 cases died among the 184 patients. The correlation between clinicopathological characteristics and the prognosis of HCC patients is shown in Table 1.

**Table 1:** Correlation between clinicopathological characteristics and the prognosis of HCC patients

Clinicopathological characteristic	Total case	Death	Univariate analysis	
			HR (95%CI)	*P*
Age (years)				
≤ 50	103	36	Ref.	
> 50	81	32	1.075 (0.858–1.348)	0.542
Gender				
Male	156	60	Ref.	
Female	28	8	0.862 (0.661–1.123)	0.397
HBsAg				
No	23	6	Ref.	
Yes	161	62	1.202 (0.916–11.578)	0.356
Tumor size (cm)				
≤ 5	76	18	Ref.	
> 5	108	50	1.421 (1.146–1.763)	0.002
Tumor number				
Single	123	15	Ref.	
Multiple	61	53	6.695 (3.497–12.82)	< 0.001
TNM staging				
Stage I–II	75	17	Ref.	
Stage III–V	109	51	1.453 (1.173–1.801)	0.001
Live cirrhosis degree				
No and mild	77	20	Ref.	
Moderate and severe	107	48	1.343 (1.082–1.666)	0.013
Differentiation degree				
Grade I–II	104	52	Ref.	
Grade III–IV	80	17	0.625 (0.501–0.780)	< 0.001

Note: HCC, hepatocellular carcinoma; HR, Hazard Ratio; CI, confidence intervals.

### Transfection efficiency of recombinant plasmids in HepG2, Bet-7402, and smmc-7721 cells

**Figure 1:**
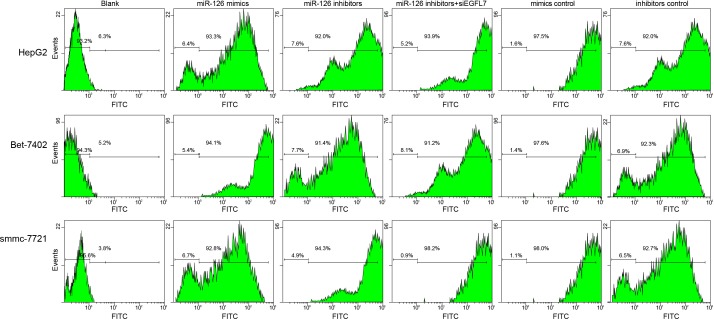
The transfection efficiency of recombinant plasmids pLEGFP-N1-miR-126 mimic, pLEGFP-N1-miR-126 inhibitor, pLEGFP-N1-miR-126 mimic NC, pLEGFP-N1-miR-126 inhibitor NC and pLEGFP-N1-si EGFL7 determined by flow cytometry A. transfection efficiency of recombinant plasmids in HepG2 cells; B. transfection efficiency of recombinant plasmids in Bet-7402 cells; C. transfection efficiency of recombinant plasmids in smmc-7721 cells; miR-126, microRNA-126; EGFL7, epidermal growth factor-like domain 7; NC, negative control.

HepG2, Bet-7402, and smmc-7721 cells were transfected with recombinant plasmids (pLEGFP-N1-miR-126 mimic, pLEGFP-N1-miR-126 inhibitor, pLEGFP-N1-miR-126 mimic NC, pLEGFP-N1-miR-126 inhibitor NC, or pLEGFP-N1-si EGFL7). The transfection efficiency up to 90% evaluated by flow cytometry indicated the successful transfection (Figure 1).

### miR-126 expression was negatively correlated with EGFL7 and ERK levels in HCC tissues and cell lines

**Figure 2:**
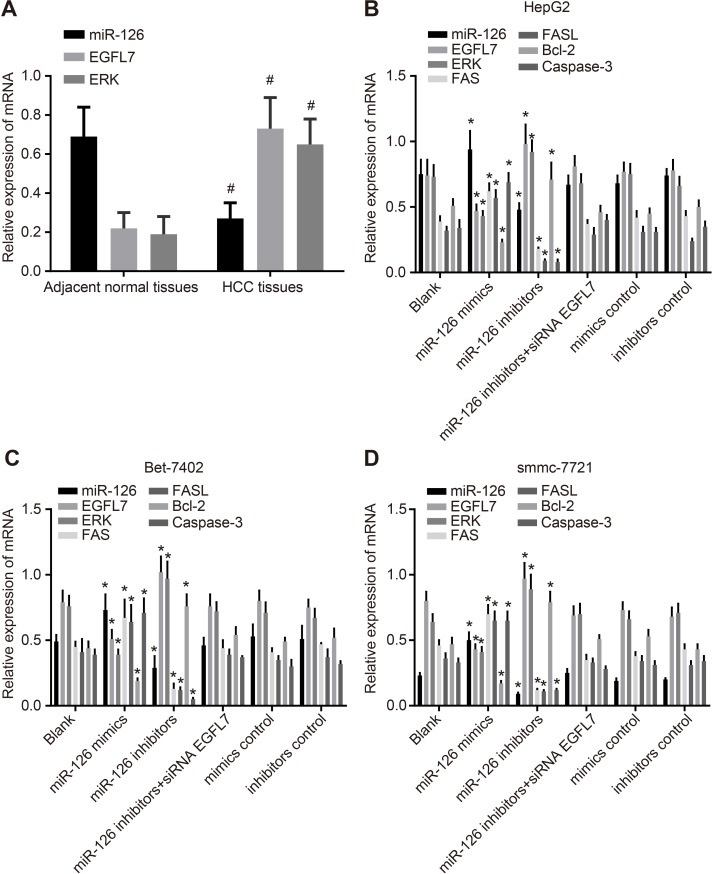
miR-126 expression and EGFL7, ERK, Fas/FasL, Bcl-2, and Caspase-3 mRNA expression in HCC tissues, adjacent normal tissues, and transfected HCC cell lines (**A**). comparisons of miR-126 expression and EGFL7 and ERK mRNA expression between the HCC tissues and adjacent normal tissues; (**B**). comparisons of miR-126 expression and EGFL7, ERK, Fas/FasL, Bcl-2, and Caspase-3 mRNA expressions in HepG2 cells among the six groups; (**C**). comparison of miR-126 expression and EGFL7, ERK, Fas/FasL, Bcl-2, and Caspase-3 mRNA expressions in Bet-7402 cells among the six groups; (**D**). comparisons of miR-126 expression and EGFL7, ERK, Fas/FasL, Bcl-2 and Caspase-3 mRNA expressions in smmc-7721 cells among the six groups; ^#^*P* < 0.05 compared with adjacent normal tissues; *P < 0.05 compared with the blank group; HCC, hepatocellular carcinoma; miR-126, microRNA-126; EGFL7, epidermal growth factor-like domain 7; ERK, extracellular signal-regulated kinase; FASL, FAS ligand; Bcl-2, B cell leukemia/lymphoma-2.

**Figure 3:**
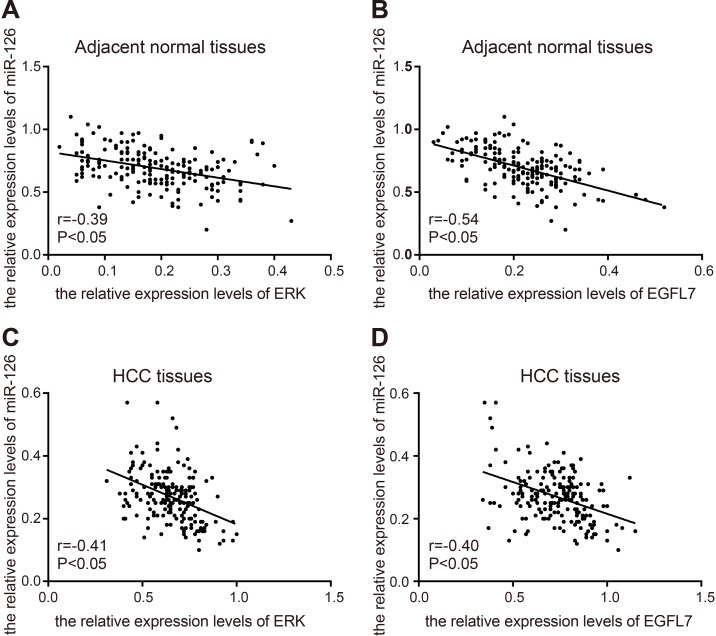
correlation analysis of miR-126, EGFL7, and ERK in HCC tissues and adjacent normal tissues (**A**). correlation analysis of miR-126 and ERK in adjacent normal tissues; (**B**), correlation analysis of miR-126 and EGFL7 in adjacent normal tissues; (**C**), correlation analysis of miR-126 and ERK in HCC tissues; (**D**), correlation analysis of miR-126 and EGFL7 in HCC tissues. r, correlated coefficient; r > 0, positive correlation; r < 0, negative correlation; miR-126, microRNA-126; EGFL7, epidermal growth factor-like domain 7; ERK, extracellular signal-regulated kinase; HCC, hepatocellular carcinoma.

Compared with the adjacent normal tissues, the miR-126 expression in HCC tissues was downregulated while EGFL7 and ERK mRNA expressions were upregulated (all *P* < 0.05), indicating low miR-126 expression and high EGFL7 and ERK expressions might promote the risk of HCC. Among three HCC cell lines (HepG2, Bet-7402 and smmc-7721), the lowest miR-126 expression was observed in smmc-7721 cells, and the highest in HepG2 cells. Compared with the blank group, no significant difference was observed in the miR-126 expression and expressions of EGFL7, ERK, Fas/FasL, Bcl-2 and Caspase3 mRNAs in the miR-126 inhibitors + si-EGFL7, mimics control and inhibitors control groups (all *P* > 0.05). In the miR-126 mimics group, the miR-126 expression and Fas/FasL and Caspase3 mRNA expressions were significantly increased and the EGFL7, ERK, and Bcl-2 mRNA expressions were notably decreased in comparison to the blank group (all *P* > 0.05). In the miR-126 inhibitors group, the miR-126 expression and Fas/FasL and Caspase3 mRNA expressions were evidently downregulated while EGFL7, ERK, and Bcl-2 mRNA expressions were markedly upregulated when compared with the blank group (all *P* < 0.05). These results showed that miR-126 expression was negatively correlated with EGFL7 and ERK (Figures 2, 3).

### Inhibition of EGFL7 blocked the ERK signaling pathway to promote the apoptosis of HCC cells

**Figure 4:**
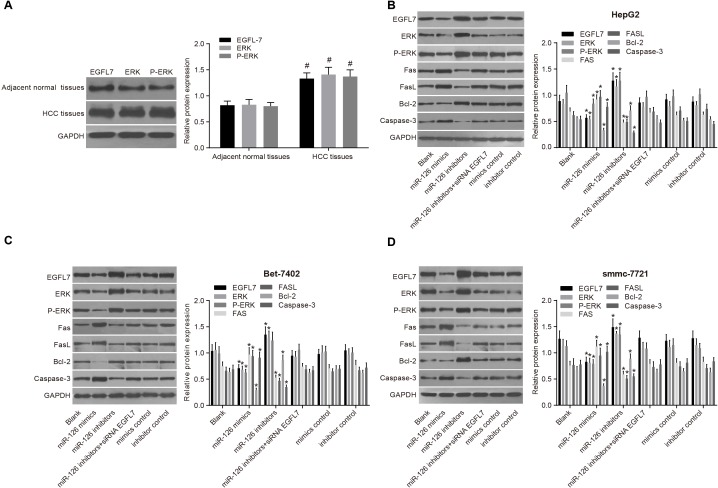
EGFL7, ERK, P-ERK, Fas/FasL, Bcl-2 and Caspase-3 protein expressions in HCC tissues, adjacent normal tissues and transfected HCC cell lines (**A**). comparisons of the expressions of EGFL7, ERK, P-ERK, Fas/FasL, Bcl-2 and Caspase-3 proteins between the HCC tissues and adjacent normal tissues; (**B**). comparisons of the expressions of EGFL7, ERK, P-ERK, Fas/FasL, Bcl-2 and Caspase-3 proteins in HepG2 cells among the six group; (**C**). comparisons of the expressions of EGFL7, ERK, P-ERK, Fas/FasL, Bcl-2 and Caspase-3 proteins in Bet-7402 cells among the six group; (**D**). comparisons of the expressions of EGFL7, ERK, P-ERK, Fas/FasL, Bcl-2 and Caspase-3 proteins in smmc-7721 cells among the six group; ^#^*P* < 0.05 compared with adjacent normal tissues; *P < 0.05 compared with the blank group; HCC, hepatocellular carcinoma; miR-126, microRNA-126; EGFL7, epidermal growth factor-like domain 7; ERK, extracellular signal-regulated kinase.

EGFL7, ERK, and P-ERK protein expressions in HCC tissues were significantly higher than these in the adjacent normal tissues (all *P* < 0.05), indicating that increased EGFL7, ERK, and P-ERK expression may contribute to the risk of HCC (Figure 4). Among three HCC cell lines (HepG2, Bet-7402 and smmc-7721), the EGFL7 protein expression was highest in smmc-7721 cells, and lowest in HepG2 cells. Compared with the blank group, no significant difference was observed in the expressions of EGFL7, ERK, P-ERK, Bcl-2 Fas/FasL and Caspase3 proteins in the miR-126 inhibitors + si-EGFL7, mimics control, and inhibitors control groups (all *P >* 0.05). The miR-126 mimics group exhibited markedly higher Fas/FasL and Caspase3 protein expressions and lower EGFL7, ERK, P-ERK, and Bcl-2 protein expressions than the blank group (all *P >* 0.05). Compared with the blank group,, Fas/FasL and Caspase3 protein expressions were decreased, and EGFL7, ERK, P-ERK, Bcl-2 protein expressions were significantly increased in the miR-126 inhibitors group (all *P <* 0.05), suggesting that the low EGFL7 expression inhibited the activation of the ERK signaling pathway, promoting the expression of pro-apoptotic factors and HCC cell apoptosis [20].

### EGFL7: the direct target gene of miR-126

**Figure 5:**
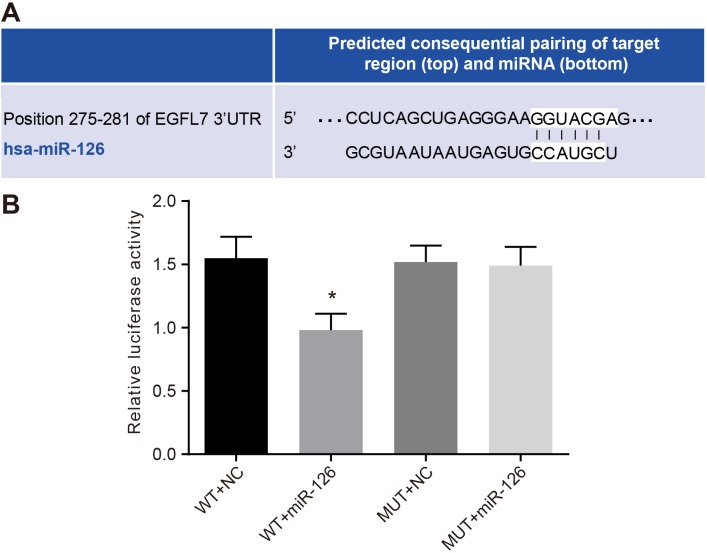
Identification of miR-126 targeting EGFL7 (**A**). the binding site of miR-126 and EGFL7 in the 3′UTR; (**B**). the luciferase activity at 48 h after the HepG2 cells were transfected with EGFL7-3′UTR-WT + NC, EGFL7-3′UTR-WT + miR-126, EGFL7-3′UTR-MUT + NC and EGFL7-3′UTR-MUT + miR-126; *P < 0.05 compared with cells transfected with EGFL7-3′UTR-WT + NC, EGFL7-3′UTR-MUT + NC or EGFL7-3′UTR-MUT + miR-126; miR-126, microRNA-126; EGFL7, epidermal growth factor-like domain 7; NC, negative control; 3′UTR, 3′ untranslated region; WT, wide type; MUT, mutant type.

The microRNA.org predicted that miR-126 was located in the introns 6 and 7 in the 3′-UTR of EGFL7 and could bind to the 3′-UTR of EGFL7 mRNA. HepG2 cells were co-transfected with EGFL7-3′UTR-WT and miR-126 mimic plasmids. Compared with the EGFL7-3′UTR-WT + NC group (Figure 5), the ratio of Firefly luciferase activity to Renilla luciferase activity (Y/H) was significantly decreased in the EGFL7-3′UTR-WT + miR-126 mimic group (*P <* 0.05), while no significant difference was observed between the EGFL7-3′UTR-MUT + miR-126 mimics and EGFL7-3′UTR-WT + NC groups (*P >* 0.05). The results indicated that EGFL7 was the direct target gene of miR-126.

### miR-126 overexpression suppressed the proliferation of HCC cells

**Figure 6:**
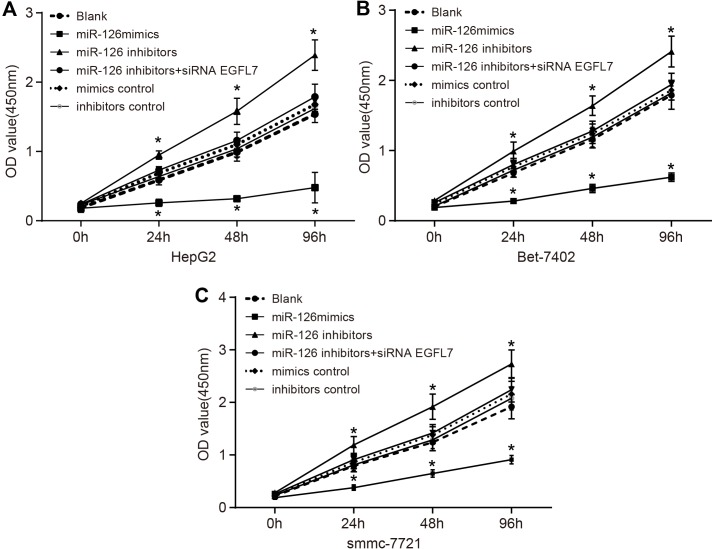
Comparison of cell proliferation among the blank, miR-126 mimics, miR-126 inhibitors, miR-126 inhibitors + siRNA EGFL7, mimics control, and inhibitors control groups after transfection (**A**). the proliferation of HepG2 cells among the blank, miR-126 mimics, miR-126 inhibitors, miR-126 inhibitors + siRNA EGFL7, mimics control and inhibitors control groups after transfection; (**B**). the proliferation of Bet-7402 cells among the blank, miR-126 mimics, miR-126 inhibitors, miR-126 inhibitors + siRNA EGFL7, mimics control and inhibitors control groups after transfection; (**C**). the proliferation of smmc-7721 cells among the blank, miR-126 mimics, miR-126 inhibitors, miR-126 inhibitors + siRNA EGFL7, mimics control and inhibitors control groups after transfection; *P < 0.05 compared with the blank group; miR-126, microRNA-126; EGFL7, epidermal growth factor-like domain 7.

Shown as Figure 6, the proliferation of smmc-7721 cells was the highest, and that of HepG2 cells was the lowest among three HCC cell lines (HepG2, Bet-7402 and smmc-7721). No significant difference was observed in the proliferation of HCC cells in the miR-126 inhibitors + si-EGFL7, mimics control and inhibitors control groups in comparison to the blank group (all *P* > 0.05). Compared with the blank group, the proliferation of HCC cells in the miR-126 mimics group was decreased while that of HCC cells in the miR-126 inhibitors group was increased significantly (both *P* < 0.05). These results indicated that miR-126 overexpression and inhibition of EGFL7 expression could suppress the proliferation of HCC cells. The proliferation of HCC cells might be enhanced when miR-126 expression was suppressed.

### miR-126 overexpression promoted the apoptosis of HCC cells

**Figure 7:**
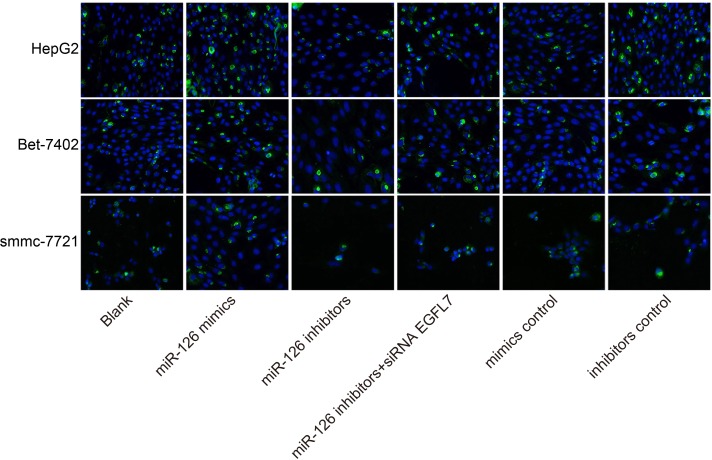
Comparisons of cell apoptosis among the blank, miR-126 mimics, miR-126 inhibitors, miR-126 inhibitors + siRNA EGFL7, mimics control and inhibitors control groups in smmc-7721, Bet-7402, and HepG2 cell lines after transfection (× 400) Note: DAPI dye cell nucleus was blue; miR-126, microRNA-126; EGFL7, epidermal growth factor-like domain 7; DAPI, 4′-6-diamidino-2-phenylindole.

After three HCC cell lines (smmc-7721, Bet-7402, and HepG2) were transfected with the recombinant plasmids, the apoptosis rate of HCC cells was significantly increased in the miR-126 mimics group and that of HCC cells was reduced in the miR-126 inhibitors group compared with the blank group, indicating that miR-126 overexpression could induce the apoptosis of HCC cells (Figure 7).

### miR-126 had no effect on cell cycle distribution of HCC cells

Flow cytometry was used to detect cell cycle distribution in the three HCC cell lines (smmc-7721, Bet-7402 and HepG2) after cell transfection (Table 2). No significant difference was observed in the ratios at G0/G1 phase, G2/M phase, and S phase of smmc-7721, Bet-7402, and HepG2 cells among all six groups (all *P* > 0.05).

**Table 2:** Cell cycle distribution in HepG2, Bet-7402 and smmc-7721 cells among the six groups

Cell cycle	Blank group	miR-126 mimics group	miR-126 inhibitors group	miR-126 inhibitors + si-EGFL7 group	Mimics control group	Inhibitors control group
HepG2						
G0/G1 phase	56.81 ± 0.23	56.79 ± 0.73	57.06 ± 0.92	56.45 ± 0.51	57.17 ± 0.33	56.98 ± 0.56
G2/M phase	12.73 ± 0.31	12.52 ± 0.42	12.96 ± 0.27	13.08 ± 0.36	12.87 ± 0.39	12.99 ± 0.44
S phase	30.46 ± 0.25	30.69 ± 0.77	29.98 ± 0.71	30.47 ± 0.78	29.96 ± 0.22	30.03 ± 0.99
Bet-7402						
G0/G1 phase	65.59 ± 1.02	64.62 ± 0.58	64.15 ± 0.62	64.30 ± 0.87	65.04 ± 1.05	64.27 ± 0.83
G2/M phase	13.38 ± 0.29	14.27 ± 0.31	13.79 ± 0.45	13.63 ± 0.38	14.16 ± 0.32	13.99 ± 0.12
S phase	21.03 ± 0.82	21.11 ± 0.80	22.06 ± 0.19	22.07 ± 0.96	20.80 ± 0.73	21.74 ± 0.71
smmc-7721						
G0/G1 phase	73.17 ± 0.62	73.85 ± 0.76	73.21 ± 0.59	74.19 ± 0.93	73.73 ± 0.61	73.68 ± 0.72
G2/M phase	7.57 ± 0.33	7.83 ± 0.41	7.67 ± 0.28	7.84 ± 0.25	8.07 ± 0.36	7.90 ± 0.19
S phase	19.26 ± 0.57	18.32 ± 0.36	19.12 ± 0.33	17.97 ± 1.15	18.20 ± 0.89	18.42 ± 0.76

Note: miR-126, microRNA-126; EGFL7, epidermal growth factor-like domain 7; si-EGFL7; siRNA EGFL7.

### miR-126 overexpression reduced the proliferation of HCC cells *in vivo*

**Figure 8:**
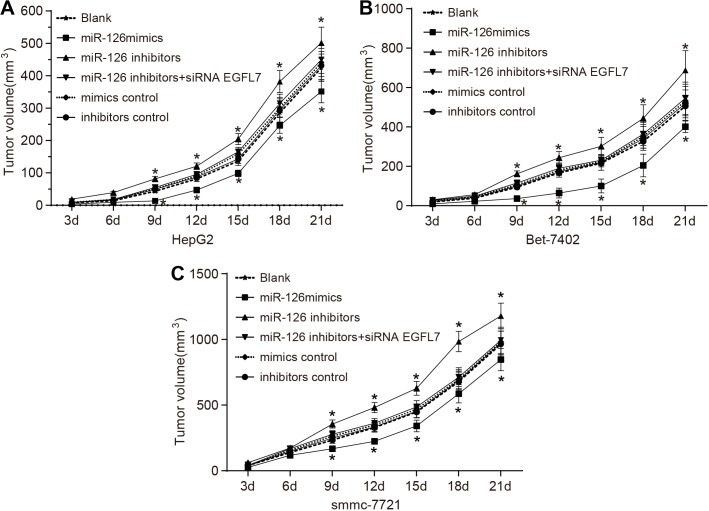
Tumor growth curves of nude mice transplanted with smmc-7721, Bet-7402 and HepG2 cell lines in the blank, miR-126 mimics, miR-126 inhibitors, miR-126 inhibitors + siRNA EGFL7, mimics control and inhibitors control groups (**A**). tumor growth curves of mice transplanted with HepG2 cells in each group; (**B**). tumor growth curves of mice transplanted with Bet-7402 cells in each group; (**C**) tumor growth curves of mice transplanted with smmc-7721 cells for each group; *P < 0.05 compared with the blank group; miR-126, microRNA-126; EGFL7, epidermal growth factor-like domain 7.

The subcutaneous tumor volume in nude mice was gradually increased three days after transplantation. The tumor formation ability of smmc-7721 cells in the nude mice was the strongest, and that of HepG2 cells in the nude mice was the weakest among three HCC cell lines (HepG2, Bet-7402 and smmc-7721). The miR-126 inhibitors group exhibited the strongest tumor formation ability with faster tumor cell growth and the miR-126 mimics group had the weakest tumor formation ability with slower tumor cell growth among the six groups (Figure 8). The mice were sacrificed after three weeks, and no significant difference was observed in tumor volume and weight of the miR-126 inhibitors + si-EGFL7, mimics control and inhibitors control groups compared with the blank group (all *P* > 0.05). The tumor weight in the miR-126 mimics group was decreased and the tumor weight in the miR-126 inhibitors group was evidently increased than that in the blank group (Table 3). Therefore, the miR-126 overexpression and inhibition of EGFL7 expression might suppress the proliferation ability of tumor cells *in vivo*. The proliferation ability of tumor cells might be enhanced by the inhibition of miR-126.

**Table 3:** Comparisons of the number of new vessels and tumor weight among the six grouos after tumor transplantation

	Blank group	miR-126 mimics group	miR-126 inhibitors group	miR-126 inhibitors + si-EGFL7 group	Mimics control group	Inhibitors control group
HepG2						
New vessels	21.13 ± 1.36	9.63 ± 3.02*	32.88 ± 3.60*	22.88 ± 3.56	20.50 ± 2.62	19.25 ± 2.12
Tumor weight	2. 41 ± 0.23	1. 04 ± 0.19*	3. 58 ± 0.34*	2. 37 ± 0.21	2. 46 ± 0.12	2. 39 ± 0.14
Bet-7402						
New vessels	28.63 ± 2.72	12.88 ± 2.30*	39.00 ± 4.90*	26.00 ± 1.85	30.00 ± 4.21	28.88 ± 3.00
Tumor weight	2.54 ± 0.15	1.23 ± 0.16*	3. 77 ± 0.31*	2.42 ± 0.22	2. 51 ± 0.27	2. 46 ± 0.30
smmc-7721						
New vessels	35.88 ± 3.98	17.00 ± 2.78*	48.00 ± 4.93*	34.88 ± 3.04	38.13 ± 4.12	33.88 ± 1.96
Tumor weight	2.76 ± 0.24	1.56 ± 0.19*	3.88 ± 0.38*	2.55 ± 0.23	2.68 ± 0.24	2.57 ± 0.27

Note: miR-126, microRNA-126; EGFL7, epidermal growth factor-like domain 7; si-EGFL7; siRNA EGFL7; *P < 0.05 compared with the blank group.

### miR-126 overexpression induced tumor angiogenesis of HCC cells *in vivo*

The number of new blood vessels was the highest in the nude mice transplanted with smmc-7721 cells, and that was lowest in the nude mice transplanted with HepG2 cells among the three HCC cell lines which were transfected with recombinant plasmids (Table 3). No significant difference in the number of new blood vessels was observed among the blank, miR-126 inhibitors + si-EGFL7, mimics control, and inhibitors control groups (all *P* > 0.05). The miR-126 mimics group had markedly less new blood vessels and that in the miR-126 inhibitors group exhibited significantly more new blood vessels than the blank group (both *P* < 0.05). Therefore, the miR-126 overexpression could inhibit tumor angiogenesis.

### miR-126 overexpression inhibited expressions of EGFL7, ERK, P-ERK, angiogenesis-associated proteins VEGF, and CD31 in HCC cells *in vivo*

**Figure 9:**
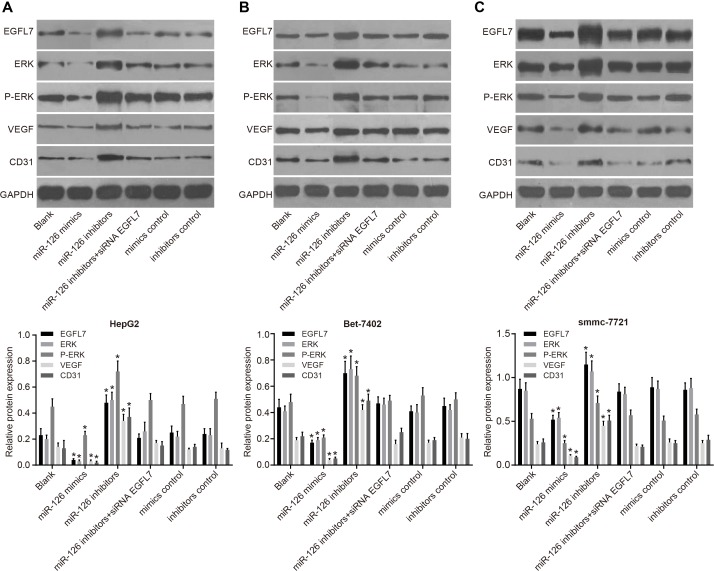
EGFL7, ERK, P-ERK, VEGF, and CD31 protein expressions in smmc-7721, Bet-7402 and HepG2 cell lines *in vivo* (**A**). EGFL7, ERK, P-ERK, VEGF, and CD31 protein expression in HepG2 cells *in vivo*; (**B**). EGFL7, ERK, P-ERK, VEGF, and CD31 protein expression in Bet-7402 cells *in vivo*; (**C**). EGFL7, ERK, P-ERK, VEGF and CD31 protein expression in smmc-7721 cells *in vivo*; *P < 0.05 compared with the blank group; miR-126, microRNA-126; EGFL7, epidermal growth factor-like domain 7; ERK, extracellular signal-regulated kinase; P-ERK, phosphorylated ERK; VEGF, vascular endothelial growth factor.

The EGFL7 expression was the highest in smmc-7721 cells *in vivo*, and lowest in HepG2 cells among three HCC cells lines which was transfected with recombinant plasmids (Figure 9). No significant difference was observed in EGFL7, ERK, P-ERK, VEGF, and CD31 protein expressions among the blank, miR-126 inhibitors + si-EGFL7, mimics control and inhibitors control groups (all *P* > 0.05). EGFL7, ERK, P-ERK, VEGF, and CD31 protein expressions were decreased in the miR-126 mimics group, but these were significantly elevated in the miR-126 inhibitors group relative to the blank group (all *P <* 0.05). These results showed that miR-126 overexpression could reduce the expression of ERK, P-ERK and angiogenesis-associated proteins, inhibiting tumor angiogenesis and tumor growth.

## DISCUSSION

In this study, we showed that miR-126 was significantly reduced in HCC tissues, and the elevation of miR-126 could decrease cell proliferation, induce cell apoptosis, and inhibit angiogenesis in HCC cells. Further experiments indicated that the function of miR-126 in HCC is achieved by targeting EGFL7 and subsequently regulating of the ERK signaling pathway.

Our initial results revealed that miR-126 is significantly downregulated, while the mRNA and protein expression of EGFL7 and ERK are upregulated in HCC tissues. MiR-126 expression is downregulated in human cancers, reducing its ability to suppress the proliferative and invasive capacities of cancer cells, such as lung cancer, osteosarcoma, oral cancer and so on [11, 12, 21]. Wong et al. have found that miR-126 is significantly decreased in patients carrying hepatitis B and C virus, and lower miR-126 expression predicts poor survival and tumor recurrence for HCC patients after surgery [22]. Also, EGFL7 is elevated in HCC patients, which functions as a chemoattractant for cell migration [23]. MiR-126 overexpression using miR-126 mimics could significantly reduce the phosphorylation of ERK protein [24]. Additionally, our study has indicated that EGFL7 is the target gene of miR-126. MiR-126 is situated in an intron of EGFL7, and miR-126 could be generated by 3 different transcripts on EGFL7 [15]. A mechanism of miR-126 targeting EGFL7 to inhibit cell proliferation has also been confirmed by Sun et al. in non-small cell lung cancer [25]. Moreover, miR-126 could reduce cell proliferation and tumor angiogenesis of HCC via decreasing EGFL7 expression [26], which is similar to our results, but the negative regulation of miR-126 on ERK signaling pathway was not involved.

Our study also demonstrates that overexpressed miR-126 decreases cell proliferation and induces cell apoptosis in HCC cells. We found that ERK and Bcl-2 mRNA and protein levels are significantly reduced in the miR-126 mimics group, while those of Fas/FasL and Caspase-3 are increased. Furthermore, the cell proliferation ability *in vivo* was reduced, while cell apoptosis was enhanced. The levels of ERK and Bcl-2 mRNA and protein were elevated in the miR-126 inhibitors group, while those of Fas/FasL and Caspase-3 were reduced. Importantly, Fas/FasL, Caspase-3 and Bcl-2 are considered as apoptosis-related genes [27]. The ERK signaling pathway is largely involved in cell proliferation and apoptosis in HCC [28–30]. Especially, the inactivation of the ERK signaling pathway could contribute to cell proliferation and induce cell apoptosis in HCC [28]. Zhao et al. have also demonstrated that the high miR-126 expression can inhibit cell proliferation, arrest cell cycle progress, and induce apoptosis in HCC cells [14], which is consistent with our study.

Moreover, overexpression of miR-126 inhibits angiogenesis triggered by the HCC cells. EGFL7, ERK, VEGF, and CD31 protein levels are reduced in the miR-126 mimics group, while EGFL7, ERK, P-ERK, VEGF, and CD31 protein are increased in the miR-126 inhibitors group. Angiogenesis is an important event in HCC progression, in which several angiogenic factors are commonly involved including VEGF and EGFL7 [31]. EGFL7 could enhance HCC metastasis and invasion by improving cell motility via the phosphorylation of focal adhesion kinase (FAK) [32]. Interestingly, EGFL7 is also reported to involve in blood vessel formation in human tumors, presenting an increase in highly vascularized tissues [25]. The blockage of ERK signaling inhibits angiogenesis in HCC with inactivated ERK1/2 phosphorylation [33]. CD31 participates in blood vessel formation during pathological processes, and CD31 expression often predicts acute and chronic angiogenesis [34]. Additionally, De Giorgio et al. have reported the loss of miR-126 may enhance angiogenesis through target specific proangiogenic factors at the *in situ* to invasive tumor transition, which could further prove our results [35].

To conclude, our study shows that miR-126 is downregulated in HCC tissues and that elevated miR-126 decreases proliferation and induces apoptosis in the HCC cells and inhibits angiogenesis. The effects of miR-126 on HCC cells may be achieved by targeting EGFL7 and down-regulating the ERK signaling pathway. However, the full understanding of miR-126 with ERK signaling in HCC is still uncertain, and more researches are still required to identify and effective targeted treatment for HCC.

## MATERIALS AND METHODS

### Ethical statement

The study was conducted in accordance with approval of the Ethics Committee of Maternal and Child Health Hospital of Hubei Province. Written informed consent was collected from each eligible patient prior to the experiment.

### Study subjects

A total of 184 patients with primary HCC who received treatment in Maternal and Child Health Hospital of Hubei Province between September 2010 and December 2013 were selected for this study. After surgery, HCC tissues and adjacent normal tissues were collected from all HCC patients, and clinicopathological data of enrolled patients were recorded. Human HCC cell lines (HepG2, Bet-7402, and smmc-7721) were purchased from the Cell Bank of Chinese Academy of Sciences (Shanghai, China). A total of 144 female nude mice (Department of Laboratory Animals, China Medical University, Shenyang, Liaoning, China) aged 3 months weighting 22 ± 4 g were selected for the study. All animal experiments in our study were in accordance with the principles of the local Laboratory Animal Care and Use, and guidelines established by the US National Institutes of Health.

### Cell culture

Cryopreserved HepG2, Bet-7402, and smmc-7721 cells were thawed in a 38°C water bath and then centrifuged at 1000 rpm for 5 min. The supernatant was discarded and the cells were cultured in Dulbecco’s Modified Eagle Medium (DMEM) medium in an incubator containing 5% CO_2_ at 37°C. The medium was replaced when cells adhered to the bottle wall. And the cells were subcultured till the cells covered 80% of the bottle bottom.

### Plasmid construction

The siRNA plasmid of EGFL7 gene (si-EGFL7) was designed based on the EGFL7 coding sequence (NP_57299) using RNAi design software (Invitrogen Inc., Carlsbad, CA, USA) (Table 4). Human mature miR-126 mimics, miR-126 inhibitors, miR-126 mimics control, and miR-126 inhibitors control were purchased from Biomics Biotechnologies Co. Ltd (Nantong, Jiangsu, China). The oligonucleotide sequences of these plasmids were synthesized with Eco RI and Bam HI endonuclease cleavage sites inserted at the two ends of sequences, and then the DNA fragment was cloned into the pLEGFP-N1 vector. Therefore, the corresponding recombinant plasmids including pLEGFP-N1-miR-126 mimic, pLEGFP-N1-miR-126 inhibitor, pLEGFP-N1-miR-126 mimic negative control (NC), pLEGFP-N1-miR-126 inhibitor NC, and pLEGFP-N1-si EGFL7. The fragment of EGFL7 gene containing miR-126 binding sites in 3′-UTR region was inserted into dual-luciferase reporter vector to construct recombinant wild-type plasmid (EGFL7-3′UTR-WT) and mutant-type plasmid (EGFL7-3′UTR-MUT). All plasmids were verified by polymerase chain reaction (PCR), restriction enzyme digestion, and DNA sequencing.

**Table 4:** Oligonucleotides sequences of si-*EGFL7* and NC

Gene	Sequence
***si-EGFL7***	5′-AGCAGATTTCCTTCCTGGA-3′
**NC**	5′-UUCUCCGAACGUGUCACGUTT-3′

Note: NC, negative control.

### Cell transfection

HepG2, Bet-7402, and smmc-7721 cells in logarithmic phase were seeded into 96-well plates (100 μL/well). And then these three cell lines were separately transfected with recombinant plasmids (pLEGFP-N1-miR-126 mimic, pLEGFP-N1-miR-126 inhibitor, pLEGFP-N1-miR-126 mimic NC, pLEGFP-N1-miR-126 inhibitor NC or pLEGFP-N1-si EGFL7) using Lipofectamine 2000 and then incubated in an incubator containing 5% CO_2_ at 37°C for 6 h. Next, cells were cultured with fresh medium containing 10% fetal bovine serum (FBS) for 48 h. Subsequently, cells were collected and washed with phosphate buffered saline (PBS) 3 times. After being fixed in 70% ice-cold ethanol at −20°C for 30 min, cells were incubated for 10 min with the addition of triton-x-100, followed by PBS washing for 3 times. The cells were re-suspended in 100 μL PBS,, after which the transfection efficiency was identified by flow cytometry. The target gene of miR-126 was identified with dual-luciferase reporter assay. HEPG2 cells in logarithmic phase were seeded into 96-well plates, and co-transfected with EGFL7-3′UTR-WT and miR-126 mimic plasmids (EGFL7-3′UTR-WT + miR-126) using Lipofectamine 2000 when the cell density was about 70%. Meanwhile, cells co-transfected with EGFL7-3′UTR-WT and NC plasmids (EGFL7-3′UTR-WT + NC) and with EGFL7-3′UTR-MUT and miR-126 mimic plasmids (EGFL7-3′UTR-MUT + miR-126) as controls. Chemiluminescence was used to detect the luciferase activity after 48-h culture in an incubator.

### Establishment of HCC nude mice model and grouping

Three transfected cell lines (HepG2, Bet-7402 and smmc-7721) were separately divided into the blank group (without transfection), the miR-126 mimics group (transfected with miR-126 mimics plasmid), the miR-126 inhibitors group (transfected with miR-126 inhibitors plasmid), the miR-126 inhibitors + si-EGFL7 group (transfected with miR-126 inhibitors + si-EGFL7 plasmid), the mimics control group (transfected with miR-126 mimics NC plasmid), and the inhibitors control group (transfected with miR-126 inhibitors NC plasmid). Transfected cells were collected and adjusted to a density of 1 × 10^6^/mL. The 12-week-old nude mice were subcutaneously injected with 1 mL of transfected HCC cells (HepG2, Bet-7402, smmc-7721) into the back of the upper limbs to ensure the comparability of results. Rats injected with the same cell line were assigned into six groups (8 mice in each group): the blank group (injection of cells without transfected plasmids); the miR-126 mimics group (injection of cells being transfected with miR-126 mimics plasmid); the miR-126 inhibitors group (injection of cells being transfected with miR-126 inhibitors plasmid); the miR-126 inhibitors + siRNA EGFL7 group (injection of cells being transfected with miR-126 inhibitors + siRNA EGFL7 plasmids); the mimics control group (injection of cells being transfected with miR-126 mimics NC plasmid); and the inhibitors control group (injection of cells being transfected with miR-126 inhibitors NC plasmid). The mice were fed in a specific pathogen-free (SPF) environment after tumor implantation, and the tumor formation in nude mice was observed. The long diameter (a) and short diameter (b) of the tumors were determined every three days. The tumor growth curve was analyzed based on the formula V = 1/2ab^2^. After 3 weeks, the mice were sacrificed and tumor tissues were isolated from them. The tumor size was calculated, and tumor tissues were preserved in liquid nitrogen for later use.

### Cell counting kit 8 (CCK8) assay

Cell lines (HEPG2, HepG2, Bet-7402 and smmc-7721) were separately seeded into four 96-well plates at a density of 10^4^/well, and added with 100 μL medium into each well. Five duplicate wells and five blank wells were set for each group. The plates were cultured in an incubator containing 5% CO_2_ at 37°C. At 0th h, 24th h, 48th h, and 96th h of culture, the CCK8 kit (10 μL) was added into each well. After 2 h of incubation, the optical density (OD) was detected by a microplate reader at the wavelength of 450 nm, and the proliferation curve was drawn. The actual OD value of each well = OD value of the test well – average OD value of the blank well.

### Terminal deoxynucleotidyl transferase dUTP nick end labeling (TUNEL) assay

Cells were added into 96-well plates (100 μL/well; 2 × 10^4^ cells/well) and incubated overnight, followed by cell transfection. Then, cells were cultured in an incubator containing 5% CO_2_ at 37°C for 48 h. The medium was discarded and cells were fixed with 4% paraformaldehyde. The cells were incubated at room temperature for 30 min and washed with PBS. After the addition of 0.3% H_2_O_2_-methanol solution, cells were incubated at room temperature for another 30 min. After PBS washing, permeabilization solution (0.1 % Triton.X-100 dissolved in 0.1 % sodium citrate solution) was added into plates for a 2-min incubation in an ice-bath, followed by PBS washing. With the addition of 50 μL TUNEL reagent, the cells were cultured in a humidified incubator at 37°C for 60 min. After three PBS washes, the cell were added with 50 μL of 4′, 6-diamidino-2-phenylindole (DAPI), followed by incubation at 37°C in the dark and another three PBS washes. Cells were observed under a fluorescence microscope and images were captured.

### Flow cytometry

Cells were seeded into 6-well plates at a density of 1 × 10^6^/well, and washed twice with PBS 48 h after transfection. Then, cells were digested with 0.25% ethylenediamine tetraacetic acid (EDTA)-free trypsin. The cells were collected in centrifuge tubes and centrifuged at 1000 rpm for 5 min. The medium was discarded and cells were resuspended in pre-cooled PBS. Next, cells were fixed using 70% ethanol and placed at 4°C overnight. After the cells were centrifuged at 2000 rpm for 5 min and washed twice with precooled PBS, 500 mL PI/Rnase was added into cells. The mixture was maintained in the dark for 45 min and transferred into flow tubes to detect the cell cycle distribution.

### Quantitative real-time polymerase chain reaction (qRT-PCR)

HCC tissues and adjacent normal tissues were added with saline solution and homogenized. At the same time, transfected cells from all groups were collected. Total RNA was extracted from the cells and tissues using Trizol method. The purity and concentration of RNA were determinated by ultraviolet spectrophotometry. The RNA integrity was assessed by agarose gel electrophoresis. Primers for miR-126, EGFL7, ERK, Fas/FasL (Fas ligand), Bcl-2 (B cell leukemia/lymphoma-2), and Caspase-3 were designed and synthesized by Shanghai Sangon Biological Engineering Technology & Services Co., Ltd. (Shanghai, China) (Table 5). Total RNA was used to synthesize cDNA by reverse transcription with the PrimescriptTM RT reagent Kit (Takara Biotechnology Ltd., Dalian, China) with a reaction system volume of 10 μL. Reaction conditions of reverse transcription: 16°C for 30 min, 42°C for 30 min, and 85°C for 10 min. qRT-PCR was performed using SYBR^®^ premix Ex Taq TM Real-time PCR Kit (Takara Biotechnology Ltd., Dalian, China). The reaction conditions of PCR were as follows: 95°C pre-denaturation for 2 min followed by 40 cycles of 95°C denaturation for 5 s, 60°C annealing for 4 s, and 72°C extension for 30 s. The OpticonMonitor3 software (Bio-Rad Laboratories, Inc., Hercules, CA, USA) was applied to analyze miR-126 expression and expressions of EGFL7 and ERK mRNAs in HCC tissues and adjacent normal tissues as well as the miR-126 expression and expressions of EGFL7, ERK, Fas/FasL, Bcl-2, and Caspase-3 mRNAs in the transfected cells. GAPDH was used as an internal control, and the relative quantitative method was applied to calculate the relative mRNA copy number (measured in triplicate). 2^−ΔΔCt^ was used to express the ratio of target mRNA expression relative to the GAPDH mRNA expression.

**Table 5:** Primer sequences for quantitative real-time polymerase chain reaction

Gene	Sequence
miR-126	F: 5′- ACACTCCAGCTGGGTCGTACCGTGAGTAAT-3′
	R: 5′- TGGTGTCGTGGAGTCG-3′
*EGFL7*	F: 5′-CAACCCGACAGGAGTGGACAGT-3′
	R: 5′-TCACGAGTCTTTCTTGCAGGAGC-3′
*ERK*	F: 5′-GGCACCAACCATTGAGCAGA-3′
	R: 5′-GATCATTGCTGAGGTGCTGTGTC-3′
*Fas*	F: 5′-GCATCTGGACCCTCCTACCTCTG-3′
	R: 5′-GACAAAGCCACCCCAAGTTAGA-3′
*FasL*	F: 5′-TTCTGGTTGCCTTGGTAGGATTG-3′
	R: 5′-ACCTTGAGTTGGACTTGCCTGTT-3
*Caspase-3*	F: 5′-TACCACGCCACCACCGGCCCA-3′
	R: 5′- GGCATTTTGGCTGTCGTCAGGGAA-3′
*Bcl-2*	F: 5′-ATGTGTGTGGAGAGCGTCAA-3′
	R: 5′-ACAGTTCCACAAAGGCATCC-3′
*GAPDH*	F: 5′-AGAAGGCTGGGGCTCATTTG-3′
	R: 5′-AGGGGCCATCCACAGTCTTC-3′

Note: miR-126, microRNA-126; F, forward; R, reverse.

### Western blotting

Tissue proteins were extracted from HCC and adjacent normal tissues of patients and tumor tissues in nude mice after lysis. Meanwhile, the total cell protein was extracted from transfected cells. The loading buffer was mixed with 20 μg of cell protein or 50 μg of tissue protein, and proteins were separated by sodium dodecyl sulfate-polyacrylamide gel electrophoresis (SDS-PAGE). These separated proteins were transferred onto a nitrocellulose membrane filter by electrotransfer, and blocked with 5% skimmed milk in PBS at room temperature for 1 h. After that, the filter containing protein samples was incubated with antibodies against EGFL7, ERK, P-ERK (phosphorylated ERK), Fas/FasL, Bcl-2, Caspase-3, VEGF (vascular endothelial growth factor), and CD31 at 4°C overnight. Subsequently, the filter was washed with PBS three times and incubated with horseradish peroxidase (HRP)-conjugated second antibody at room temperature for 1 h. After three PBS washes, protein samples were developed by the enhanced chemiluminescence method. Images of protein bands were captured by Gel imaging system (Bio-Rad Laboratories, Inc., Hercules, CA, USA). The gray-scale value of protein bands was analyzed with β-actin as the internal reference. The ratio of gray-scale values between the target protein band and the internal reference band was calculated as the relative protein expression.

### Determination of tumor angiogenesis

The tumor-transplanted nude mice were intravenously injected with 1% Evans Blue. Later, the mice were sacrificed and tumor tissues were isolated from them. The number of vessels increased in the direction of tumors was counted under dissecting microscope. The experiment was conducted with modified method on the basis of the investigation of Kreisle and Ershler [36].

### Dual-luciferase reporter gene assay

The targeting gene (EGFL7) of miR-126 was predicted using microRNA.org (http://www.microrna.org) and verified by dual-luciferase reporter gene assay. The luciferase activity (Firefly luciferase and Renilla luciferase) was detected according to the manufacturer’s protocol (Promega Corp, Madison, Wisconsin, USA). The medium in the 96-well plate was removed and cells in the medium was lightly washed with 100 μL PBS. After the addition of 100 μL passive lysis buffer (PLB) into each well, cell lysis buffer was collected after gently shaking at room temperature. The cell lysis buffer (20 μL) was mixed with luciferase detection agent II (100 μL), after which the Firefly luciferase activity was obtained using a chemiluminescence detector. Subsequently, the mixture was added with 100 μL of 1 × Stop & Glo Reagent to detect Renilla luciferase activity. Reporter gene expression was expressed as the ratio of Firefly luciferase activity to Renilla luciferase activity.

### Follow-up

The clinical data and follow-up information of all patients were collected. The prognostic information of patients was collected through referral, re-admission records and regular telephone interviews and other ways. The follow-up was ended up in March 2016.

### Statistical analysis

All data were analyzed using SPSS 21.0 statistical software (SPSS, Chicago, IL, USA). Measurement data are expressed as $\bar{\text{x}}\pm \text{s}$. Comparisons of measurement data between two groups were conducted using *t* test and these among multiple groups were tested by the one-way analysis of variance (ANOVA). Comparisons of enumeration data among groups were performed using the χ^2^ test. Univariate analysis was used to analyze the correlation between clinicopathological characteristics and the prognosis of HCC patients. And the correlation analysis of protein expression was analyzed by Pearson correlation analysis. *P* < 0.05 was considered to indicate statistical significance.
